# Profiling the AI speaker user: Machine learning insights into consumer adoption patterns

**DOI:** 10.1371/journal.pone.0315540

**Published:** 2024-12-18

**Authors:** Yunwoo Choi, Changjun Lee

**Affiliations:** 1 Institute of Interaction Science, Sungkyunkwan University, Seoul, South Korea; 2 School of Convergence, College of Computing and Informatics, Sungkyunkwan University, Seoul, South Korea; Federal University of Goias: Universidade Federal de Goias, BRAZIL

## Abstract

The objective of this study is to identify the characteristics of users of AI speakers and predict potential consumers, with the aim of supporting effective advertising and marketing strategies in the fast-evolving media technology landscape. To do so, our analysis employs decision trees, random forests, support vector machines, artificial neural networks, and XGboost, which are typical machine learning techniques for classification and leverages the 2019 Media & Consumer Research survey data from the Korea Broadcasting and Advertising Corporation (N = 3,922). The final XGboost model, which performed the best among the other machine learning models, specifically forecasts individuals aged 45–50 and 60–65, who are active on social networking platforms and have a preference for varied programming content, as the most likely future users. Additionally, the model reveals their distinct lifestyle patterns, such as higher internet usage during weekdays and increased cable TV viewership on weekends, along with a better understanding of 5G technology. This pioneering effort in IoT consumer research employs advanced machine learning to not just predict, but intricately profile potential AI speaker consumers. It elucidates critical factors influencing technology uptake, including media consumption habits, attitudes, values, and leisure activities, providing valuable insights for creating focused and effective advertising and marketing strategies.

## 1. Introduction

The Internet of Things (IoT) technology serves as a pivotal force in shaping the hyper-connected society engendered by the Fourth Industrial Revolution. Owing to the widespread adoption of mobile devices, such as smartphones and tablet PCs, IoT technology has permeated diverse facets of human life. It has garnered significant attention across various sectors, including automotive, agriculture, and energy. Within the context of the Fourth Industrial Revolution, IoT is exerting a transformative impact on businesses, governmental agencies, and consumers alike.

IoT technology holds relevance not merely for industrial applications but also for consumer engagement. As more IoT applications are connected and interoperated together, individuals can enjoy a richer user experience through interactions between them [1]. Artificial Intelligence (AI) speakers are among the most popular of these products [2]. Defined broadly, AI speaker is a device that utilizes artificial intelligence algorithms, such as natural language processing, to interpret and execute voice commands from the user [3]. Since the launch of Amazon’s Echo in late 2014, numerous AI speaker products have been introduced by leading IT companies, including Google’s "Google Home" in 2017 and Apple’s "Home Pod" in 2018. A report from [4] suggests that the global smart speaker market is poised for a Compound Annual Growth Rate (CAGR) of 17.1%, potentially reaching $21.6 billion by 2027. AI speakers’ assistive capabilities and user-friendly technology can help not only the average consumer, but also the disabled, the elderly, and other marginalized groups. Nevertheless, as a consumer IoT product, the AI speaker market is showing signs of stagnation. This trend raises questions about the long-term market prospects for IoT products, such as AI speakers.

Most consumer studies on IoT are theoretically anchored in the Technology Acceptance Model (TAM) or derivatives thereof. However, these studies often fall short in capturing the nuances of consumer behavior and decision-making processes. In the domain of advertising and marketing, machine learning algorithms are deemed highly valuable, yet their application has been minimally explored in IoT consumer research within the social sciences.

Consequently, the present study endeavors to offer pragmatic solutions to the industry through an academic lens by incorporating machine learning algorithms as the primary research methodology. Utilizing publicly available consumer data, which encompasses a wide array of consumer information, this study aims to develop a predictive model for AI speaker adoption. It seeks to identify key predictors and formulate a consumer profile that is likely to embrace this technology.

The primary objective of this study is to identify the characteristics of potential consumers of AI speakers, with the aim of supporting effective advertising and marketing strategies in the fast-evolving media technology landscape. Consumer behavior is not confined to a specific target but is influenced by an amalgamation of factors including demographic traits and consumption values. Accordingly, this study focuses on the wealth of consumer contextual data that can influence the adoption of AI speakers, which are representative IoT products. We aim to construct a predictive model for AI speaker adoption, identify its key predictors, and characterize the potential consumers of these devices.

## 2. Literature review

### 2.1. IoT and AI speaker

Recently, the fourth industrial revolution has emerged, integrating the physical world with the information age. The Industrial Internet of Things (IIoT) is driving this fourth industrial revolution [5]. The IIoT is the use of IoT in industries and applications that connect the physical world of sensors, devices, and machines with the Internet and use software to transform vast amounts of data into new insights and intelligence [5].

IIoT has been implemented in the area of environment monitoring, agriculture, construction industries, disaster management, solar assisted systems, robotics technology, health care, automotive industries, emergency response systems, supply chain management systems, transportation and many more [6,7]. First of all, with regard to public safety, IIOT continues to grow as a promising network paradigm for monitoring radioactive contamination levels, which pose a great threat to public health and environmental protection [8]. In public emergencies, deep learning applications based on IoT-native big data can be utilized to predict and manage dynamic changes in Online Public Sentiment [9]. Furthermore, while IIOTs already provide a variety of services such as traffic management, road safety, and data sharing, [10] proposed a novel methodology to ensure data security. In particular, during the COVID-19 pandemic, the demand for intelligent health surveillance and diagnosis systems has been increasing globally [11]. As a result, attempts to integrate AI and IoT in the healthcare sector are being realized, including diagnostic systems for patients with severe heart disease [11], urinary tract infection detection systems [12], and health monitoring systems for patients with autism spectrum disorders [13].

IoT is not only penetrating the industrial sector, but also into the daily lives of consumers. IoT has boosted the automation and remote control that enable the monitoring and steering of the devices used in modern households [14]. The so-called ‘smart home’ is considered a promising area for IoT applications because companies with core wireless network technologies can provide smart home platform solutions. IoT technologies in smart homes can provide economic benefits and improved living by integrating with smart grid systems and enabling easy access to wireless networks [15].

Currently, consumer spending on the smart home sector is increasing globally [16]. Smart home technology also supports a wide range of smart devices, lighting, and other appliances. AI speakers are particularly popular, with 31% of U.S. households reportedly using them [17]. AI speaker is a type of consumer IoT device designed to interact with users through voice commands. Typically, this device is connected to the internet to access vast amounts of information and perform various tasks in real time, and is equipped with advanced artificial intelligence and speech recognition technology to understand natural language and respond to user requests [18]. It can also integrate with other devices and services, such as music streaming services, smart home systems, and virtual assistants, to provide more versatility. Overall, AI speakers have the advantage of giving people real-time access to information and easy control of their devices without the need for physical contact [19]. Moreover, the ability of AI speakers to assist humans can create opportunities to maintain, improve, or promote the functional abilities of people with disabilities and the elderly, as well as the convenience of the general consumer [20]. From this perspective, the mass adoption of consumer IoT products such as AI speakers could provide a good ratio of social return on investment by lowering the cost threshold [20].

Nevertheless, consumer IoT deployments consist only of few devices compared to IIoT networks [21]. Notably, the use of AI speakers is not very popular in some countries [18]. A decline in consumer engagement with AI speakers has been reported, as users have either ceased using the devices or are utilizing only a subset of their functionalities [22]. Therefore, a comprehensive understanding of users is crucial for the successful diffusion and sustained use of AI speakers as a representative consumer IOT product [19]. Furthermore, efforts to identify potential consumers based on this understanding are needed.

### 2.2. The potential of machine learning algorithms as methodologies in consumer studies for IoT products

Consumer research on Internet of Things (IoT) products can be broadly classified into four principal categories. First, a predominant number of studies focus on the elements influencing consumer acceptance of IoT and are largely grounded in models derived from the Technology Acceptance Model (TAM) and the Unified Theory of Acceptance and Use of Technology (UTAUT) [23–27]. Second, certain investigations examine the impact of individual factors on consumers’ receptivity towards IoT, further verifying the effect of salient characteristics of IoT products, as adopted from TAM, on consumers’ purchasing intentions and usage attitudes [28–30]. Third, emerging scholarship endeavors to conceptualize models that delineate consumer acceptance of IoT [31,32]. Fourth, [15] employ Peter Mobile’s UX Hive model to assess consumer satisfaction with location-based IoT services on smartphones, accounting for class-based differences.

The acceptance of AI speakers, which is the main focus of our study, is also dominated by studies based on TAM and TAM-derived models as their theoretical foundation. In terms of TAM, perceived ease of use and perceived usefulness are considered to be strong influencers of smart speaker acceptance, while perceived enjoyment, the quality and diversity of a system, level of security, and price have been reported as additional influential factors in the extended version of TAM [33–35]. Among the studies based on UTAUT2, [36] identified performance expectancy, price value, and habit conditions were important variables in adopting AI speakers, while [37] argued that effort expectancy, habit, social influence, and hedonic motivation positively influence their acceptance. [38] conducted a study based on the Theory of Reasoned Action that underlies the development of these technology adoption theories and models, and their results show that perceived risk, privacy concerns, and perceived learnability of smart speakers influence consumers’ intention to use smart speakers for voice shopping, which in turn influences actual use.

Notwithstanding their contributions, existing models, principally TAM—originally proposed by [39]—and its derivatives like UTAUT, UTAUT2, and E-TAM by [40], are not without limitations. These models, employing "descriptive" statistical methodologies such as multiple regression analysis and structural equation modeling, focus on relationships between determinants of technology use [41]. Notably, Partial Least Squares (PLS) structural equation modeling, commonly utilized in TAM and UTAUT research, restricts the number of potentially significant influential constructs that can be incorporated into an analysis [42]. Consequently, these models often overlook factors pertaining to social change processes and innovation [43].

Furthermore, the orientations of TAM and UTAUT predominantly emphasize consumer behavior rather than the ultimate objectives of technology usage, thus neglecting key motivators of user behavior and emotional engagement [44,45]. This limitation renders these models ineffective in providing actionable insights for practitioners to identify nuanced patterns in consumers’ usage and decision-making [43]. Specifically, [46] highlight the inadequacy of TAM in accounting for the acceptance of new Information and Communication Technologies (ICTs), suggesting that users should be perceived as ’consumers’ of technology products rather than mere adopters. Furthermore, given the rapid advancements in digital technologies, the consumer decision-making landscape is evolving in complex ways that extant models struggle to encapsulate [47]. In this evolving context, scholars anticipate the necessity for more dynamic models to better capture the intricacies of consumer behavior [48].

The limitations of traditional statistical models can be partially addressed using machine learning techniques, which allow for the integration of extensive survey data without the need for arbitrary transformation or combination [49]. Machine learning algorithms are not bound by strict statistical assumptions, enabling them to offer predictive accuracy scores that leverage the maximum amount of available data to forecast individual consumer behavior.

Machine learning is a subset of artificial intelligence that employs mathematical algorithms to learn from data, aiming to understand specific phenomena. It is a data-driven methodology where the algorithm self-learns to analyze extensive datasets, subsequently deriving a set of rules to construct a predictive model for new information [41]. Machine learning models gauge the predictive power by calculating the average difference between observed and predicted data. For instance, a machine learning-based regression model does not outright reject a hypothesis but rather estimates a target value based on the collected data [50]. However, implementing machine learning techniques is not without challenges. High error sensitivity remains a significant issue, as errors in the algorithm can lead to substantial problems [51]. Additionally, the interpretability of results generated by machine learning models is often a concern, as the decision-making processes of these algorithms are not readily transparent and may be difficult to understand [52]. Despite these hurdles, [51] anticipate that advancements in the field may soon overcome some of these current challenges.

### 2.3. Leveraging consumer data to identify potential customers for IoT products

In an ever-changing external environment, where consumer consciousness and behavior are in constant flux, understanding the contextual factors surrounding consumers is crucial for effective market segmentation and analysis. Nowadays, user data—including lifestyle, hobbies, interests, purchasing behavior, and demographic factors—collected from various online and offline customer touchpoints, serves as a vital resource for advertising and marketing strategies. Big data significantly enriches the information available to advertisers and marketers about consumers [48]. In particular, third-party data enhances their ability to acquire desired user profiles for new customer acquisition [53]. While first-party data offers a narrower scope capturing user behavior on specific channels, third-party data expands the volume and breadth of targeting, thus improving the accuracy of user profiling. Within the realm of user-based targeting, look-alike targeting has emerged as an effective strategy for audience expansion. It identifies and targets individuals who exhibit behaviors and characteristics similar to those of the core audience, based on commonalities such as demographics, lifestyle, interests, and values [54,55].

Unfortunately, little research has been conducted on the consumer characteristics that influence the acceptance of IoT products. Since IoT products are new technologies, this study reviews the literature on factors related to the acceptance of new technology products and factors that influence consumption decisions.

First, consumption value has been validated as a predictor of mobile technology adoption by several studies. [56] argued that hedonic and emotional values are better predictors of mobile auction acceptance than functional and social values. [57] analyzed the direct impact of the consumption value dimensions of price, functionality, social, emotional, situational, and novelty on the intention to use location-based mobile services. [58] found that the consumption value model predicted mobile banking acceptance among Israelis.

The second is impulsivity and behavioral propensity. Recently, many researchers in the consumer and marketing fields have argued that irrational consumption tendencies should be considered as an important factor that can influence consumption [59]. The relationship between users’ personal dispositions or consumption behaviors and their use (or reaction) to new technologies has long been studied in the literature. Many studies have explored the relationship between individual impulsivity and excessive ICT use [60,61] argue that consumers’ inherent impulsivity is an important factor in understanding how and why consumers react impulsively to website quality. [62] found that the relationship of cell phones, a new media at the time of the study, was consistent with adolescents’ consumption styles: addictive use of cell phones was associated with a trendy and impulsive consumption style, and values such as passion for technology and being trendy were also associated with an impulsive consumption style. In contrast, moderate cell phone use was associated with environmentalism and frugality.

Third, [63] argued that lifestyle is a way of living or the way one spends time and money and is an important human characteristic that influences consumption behavior. [64] argued that lifestyle differentially affects smartphone purchase motivation. [65] argue that lifestyle orientation leads to differences in cell phone usage. [66] examined the acceptance of seven technologies in China and found that lifestyle significantly influenced the differences between adopters and non-adopters.

As the fourth factor, media use has great potential as a key predictor of new technology adoption. Empirical studies have found that media use may differ between adopters and non-adopters of new technologies. [67] found that the willingness to adopt digital TV in the United States was related to time spent on the internet. More recently, [68] empirically tested the spillover effect of internet usage on intention to use IoT, demonstrating the possibility that different media usage can affect the acceptance of IoT products.

Based on the above discussion, it is important to predict the characteristics of potential consumers based on a comprehensive understanding of AI speaker users’ product acceptance factors in order to expand the consumer IoT market. Therefore, from a lookalike targeting perspective for audience expansion, this study aims to identify the characteristics of AI speaker users and predict potential consumers. To do so, we utilize third-party consumer data that is effective for market segmentation and understanding the context surrounding consumers, while using various machine learning algorithms as analytical techniques and comparing their performance as a powerful predictive methodology. Based on this objective, we propose the following research questions:

**RQ 1:** What are the characteristics of AI speaker users identified through machine learning techniques?
**RQ 1–1:** Which predictive models are most effective at forecasting AI speaker
adoption when using machine learning techniques?
**RQ 1–2:** What are the key predictors of AI speaker adoption as predicted by machine
learning techniques?
**RQ 2.** What are the characteristics of the potential consumers of AI speakers profiled by a machine learning model?

## 3. Method

### 3.1 Data

This study employs data from the 2019 Media & Consumer Research (MCR) survey conducted by the Korea Broadcasting and Advertising Corporation (KOBACO) to predict AI speaker adoption. The MCR is an annual nationwide marketing survey in South Korea that has collected data on consumer media usage and behavior from 1999 to 2019. MCR survey consists of various questions about the demographic characteristics, lifestyle, media usage behavior, and product usage behavior of media audiences, and is used for various purposes such as establishing marketing data and advertising strategies for companies and advertising companies.

MCR survey data as a secondary data source has the possibility that researchers may not fully recognize the problems of related data by not directly participating in the research process [69]. Nevertheless, in general, public data held by the government or public institutions are recognized as relatively more reliable than individually acquired data, as they are used as the basis for implementing government policies in developed countries such as the United States and the United Kingdom [70]. In particular, MCR data, which has large-scale data in terms of survey subjects, survey questions, and years of implementation, has a high research value as a source of information that allows random sampling, which is very important in terms of securing the representativeness of the sample and the validity of the research results, provided that the premise that it contains enough information to meet the research purpose is met.

The 2019 MCR was conducted as a face-to-face, individual interview survey between July 15 and September 6, 2019. A total of 4,000 men and women aged 13 to 64 were sampled through cluster sampling and quota sampling based on gender and age proportionality. Of the total 4,000 respondents, 3,922 data were used in this study, excluding 68 respondents with missing data. Of the total 3,922 respondents, 321 were AI speaker users and 3,611 were non-users.

### 3.2 Procedure for deriving variables

Out of the total 3,623 questions in the 2019 MCR, the study targeted all variables related to demographic characteristics and acceptance of new technology products; consumption values and Impulsivity and Behavioral Tendencies; media use; and lifestyle-related measures. To do so, two Ph.D students specializing in advertising and marketing were used as researchers to conduct the first round of data collection. A total of 138 questions were selected from the primary survey, including 30 questions on consumption values, 5 questions on impulsivity and behavioral tendencies, 36 questions on lifestyle, 65 questions on media usage attitudes, and 1 question on whether or not to use the dependent variable, AI speakers.

In order to ensure the content validity and reliability of the measured variables, we conducted additional variable evaluation though academic and industry experts. two Professors, a Ph.Ds and four industry professionals in advertising and marketing were selected as the criteria. The experts’ evaluations were conducted individually via email in two rounds over the course of about a week. All experts were informed about the purpose of the evaluation, the voluntary nature of their participation, and their right to withdraw at any time. We got informed consent from all of our experts by handing them a consent form and having them sign to agree to the consent form before going into the assessment. In the first round, 95 of the 138 total questions received variable validity from all experts. For the remaining 43 items, we sent each expert a copy of the opinions of the other six experts to share and reconcile their views, and then asked them again whether they wanted to include variables. As a result, more than six experts agreed that 17 out of 43 questions were appropriate as measurement variables, and 112 questions were finally selected as measurement variables. The final set of variables consisted of 26 consumption values, 5 impulsivity and behavioral tendencies, 22 lifestyle, 57 media time and attitudes, and 1 question on the dependent variable, AI speaker usage (see S1 Appendix).

### 3.3 Ethics statement

This study was conducted in accordance with the principles outlined in the Declaration of Helsinki. First, all participants provided informed consent via email prior to their participation in the expert assessment. We then obtained additional handwritten, signed consent forms from participants using the PLOS Journal form.

### 3.4 Independent variables

#### 3.4.1 Consumption value

A total of 26 items were included in the list of 10 sub-dimensions that make up the five consumption values. Product attribute value was made up of four utility-oriented items and two safety-oriented items, but personal-oriented value consisted of two autonomy-oriented items and three self-expression-oriented items. Other-oriented value was composed of two display-oriented items, while consumption community-oriented value included three social justice-oriented items and five eco-friendly-oriented items. Lastly, consumer subjective value splits over two autonomy-oriented items and three self-expression-oriented items. All the items belonging to consumption values were measured on a 6-point Likert scale ranging from ‘① Not at all’ to ‘⑥ Very much’.

#### 3.4.2. Impulsivity and behavioral tendencies

Impulsivity and behavioral tendencies were measured by a binary classification of ‘rational consumer tendencies’ and ‘irrational consumer tendencies’. Rational tendencies consisted of a total of 5 statements related to unplanned consumption, including sufficient information-seeking behavior before purchase and comparison of the utility of goods and services. Like consumption value, items related to impulsivity and behavioral tendencies were designed to respond on a 6-point Likert scale from ‘① not at all’ to ‘⑥ very much’.

#### 3.4.3. Lifestyle

The scales related to lifestyle are divided into four categories: leisure activities, life values, attitudes toward new technology, and life satisfaction. Leisure activities were converted from three existing items that individually listed the leisure time required for each day of the week, such as weekdays, Saturdays, and Sundays, into ‘average weekday leisure time’ and ‘average weekend leisure time’. Second, three items measuring ‘alone leisure orientation’ were placed. Life values consisted of seven items measuring ‘tradition/Confucianism’, ‘individualism’, and ‘hedonism’. Attitudes toward new technology consisted of five items measuring ‘understanding and favorability toward 5G’, and the last item measuring life satisfaction was also measured by five statements. Except for one item ‘weekly leisure time’ that was recorded in minutes, the remaining 21 items were measured on a 6-point Likert scale from ‘① not at all’ to ‘⑥ very much’.

#### 3.4.4. Media usage and attitude

Variables for media usage and attitude split over media usage amount, attitude towards media, and SNS activity. In the media usage amount question of MCR, the weekday, Saturday, and Sunday usage times of eight media such as terrestrial broadcasting, comprehensive programming channels, cable TV, radio, and DMB are recorded respectively. In this study, the Saturday and Sunday usage times were combined and analyzed by converting them into “weekday usage time” and “weekend usage time”. Next, the attitude towards the media is measured by the degree of entertainment value, reliability, fairness, informativeness, and selectivity that respondents feel about each medium. Respondents are asked to respond on a 5-point Likert scale from “① not at all” to “⑤ very much”. Finally, regarding SNS, there is a question about weekly usage frequency.

### 4.4 Dependent variable: Acceptance of AI speakers

In this study, we analyze the acceptance of AI speakers, which are representative products of the Internet of Things, to identify research problems 1 and 2. We converted cases where the product is used to ‘1’ and cases where it is not used to ‘0’ respectively as categorical variables and used them as dependent variables for each analysis.

### 4.5. Models

In this study, decision trees, random forests, support vector machines, artificial neural networks, and XGboost, which are typical machine learning techniques for classification or prediction, were used in the analysis. After deriving the optimal model for each of the five machine learning algorithms through 10-fold cross validation, we compare the performance of the models and finally selected one machine learning model with the best performance as the prediction model of AI speaker acceptance.

Decision tree is a technique to represent the results of recursive partitioning, which divides data into subsets according to partitioning rules based on actual decisions or probabilities of occurrence, in a tree structure. The goal is to find patterns and rules in the data by partitioning the nodes consisting of variables to form a tree. It is a method of machine learning that shows which bifurcations of conditions lead to optimal decisions. The structure of the tree starts from the root node, which is the top node that contains all the data sets, and gradually extends from the parent node to two or more child nodes, which is called the splitting method. The lowest node that no longer splits is called a leaf node.

Random forest is a popular ensemble technique that combines bagging and decision tree models, using the bootstrap method to generate a large number of samples and applying a decision tree model to synthesize the results. It was developed by [71] to compensate for the overfitting problem of decision trees. Bagging, which stands for Bootstrap Aggregation, creates n bootstrap sets with the same number of data as the original data through restoration extraction from original data to create each decision tree model. In other words, random forests randomize not only observations but also variables in the process of forming multiple tree models to form uncorrelated trees and adopt the results predicted by the majority of trees. As the model building process reduces the correlation between the decision trees, the forecast error is reduced [71].

A support vector machines is an algorithm that finds the optimal decision space by finding a hyperplane that increases the distance between two classes and creates a very homogeneous split on either side. In other words, the algorithm finds the boundary with the greatest width between the different data among the multiple boundaries that divide the data. The basic idea of this algorithm is to learn how to divide the two groups by measuring the distance between the data in each of the two groups to be classified, finding the center between the two data, which is called the decision boundary, and finding the optimal hyperplane that maximizes the margin in the center. If it can be divided by a straight line, a linear classification model is applied; otherwise, a non-linear classification model is used. The support vectors are the observations that are located a margin away from this max-margin hyperplane, and by definition, if the position of the support vectors changes, the max-margin hyperplane will also change. On the other hand, the hypersurface that serves to separate the dataset has the form of N-1 dimensions in N dimensions. For example, objects captured on a plane in two dimensions are separated by a one-dimensional subspace, a straight line, which separates them into two populations, each bounded by a straight line.

An artificial neural network is a statistical learning algorithm that predicts the optimal output value for an input value through data-driven iterative learning by artificial neurons that form a network of synaptic connections like the human brain [72]. In general, an artificial neural network consists of an input layer, a hidden layer, and an output layer. Each layer is composed of one or more nodes, which are connected and interact with the nodes in the other layers with certain weights. Each node in the input layer is connected to each neuron in the subsequent hidden layer, which in turn is connected to the output layer. The input layer consists of nodes corresponding to each input variable, and the hidden layer consists of multiple hidden nodes. In the case of the hidden layer, it is responsible for processing the linear combination of variable values received from the input layer into a nonlinear function and passing it back to the output layer or another hidden layer. The output layer refers to the nodes corresponding to the output variable, and in the case of a nominal target variable, it creates as many output nodes as there are classes.

A representative algorithm using boosting methods, the XGboost algorithm stands for Extreme Gradient boosting. XGboost utilizes an objective function consisting of a loss function representing the difference between predicted and actual values and a complexity function of a tree model to create an optimal model. It has the advantage of reducing the variance in the overall model and improving the predictive power by combining multiple classifiers with relatively low predictive power into a classifier with relatively high one [73].

### 4.6 Analysis procedure

**Fig 1:**
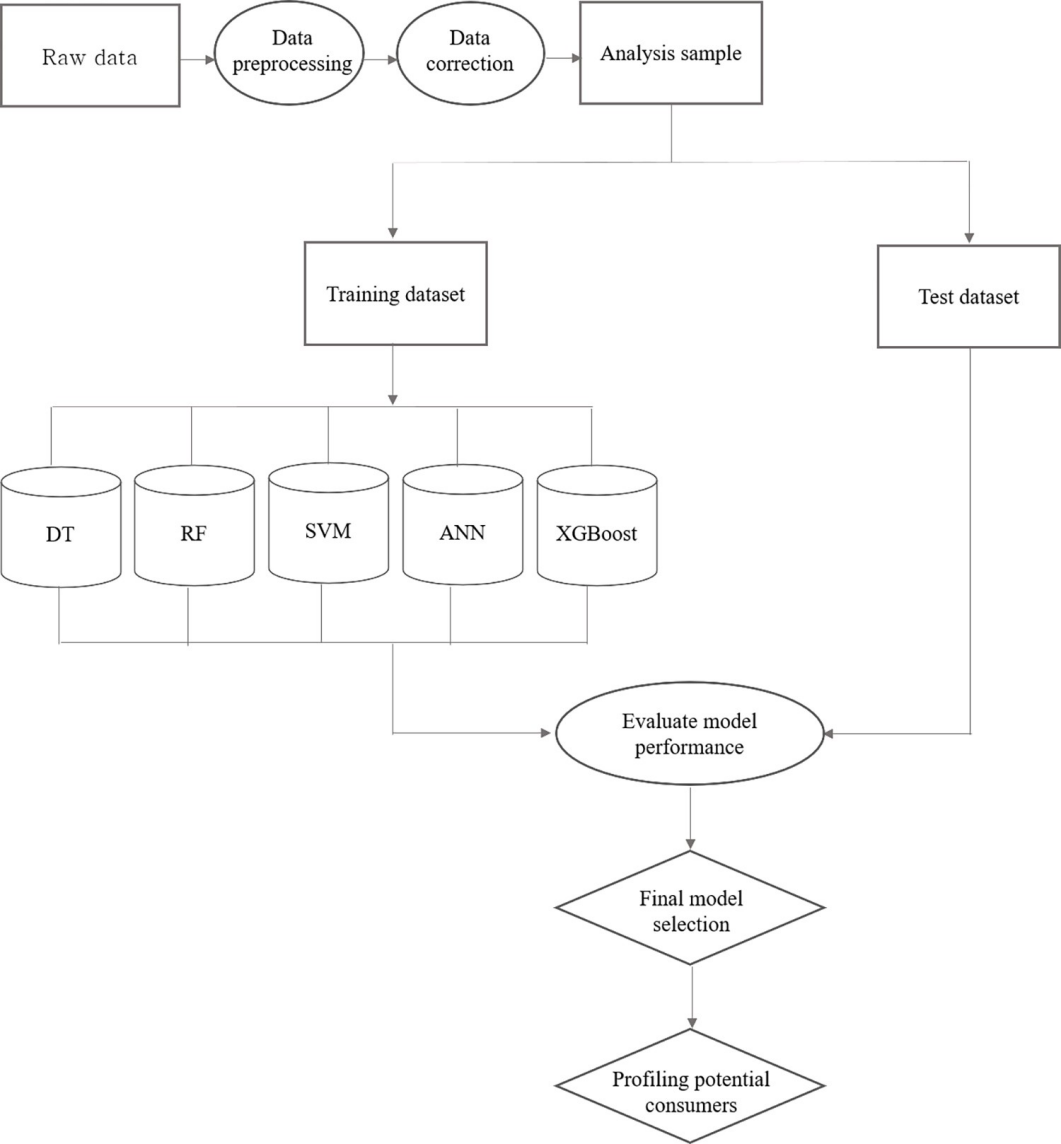
Analysis procedure process.

This paper aims to develop a predictive model of IoT adoption based on consumer contextual data and empirically verify whether there are latent predictors not captured by existing theories and models. To this end, this study was conducted in four stages: data preparation, model generation and selection, model evaluation and final selection, and potential consumer profiling. The process of building a predictive model is shown in Fig 1 below.

## 5. Results for AI speaker acceptance factors

### 5.1. Descriptive statistics & analytic sample

As shown in Table 1, out of a total of 3,922 people, there were 321 users and 3,611 non-users of AI speakers. AI speaker users are 53.9% male and 46.1% female, while non-users are 50.3% male and 49.7% female. The age composition of the user group is dominated by those in their 20s (28.35%) and 30s (28.04%). The non-user group has a higher proportion of older users, with the highest proportion in their 40s (21.26%) and 50s (21.41%). The educational attainment of AI speaker users is 67.60% college, 26.8% high school, 2.80% elementary and middle school, and 2.80% graduate school, while the non-user of AI speaker is 50.40% college, 37.97% high school, 8.89% elementary and middle school, and 2.63% graduate school. The occupations of AI speaker users include office workers (24.30%), students (20.87%), and sales/service workers (20.56%). In contrast, the non-user group’s occupational distribution is more evenly distributed, with all occupations in the 10% range, except for administrative/professional and no occupation/etc. The largest difference in composition between the two groups is marital status. While the user group has a similar mix of married (52.65%) and single (46.11%) people, the non-user group is overwhelmingly made up of married people (63.94%) over single people (32.51%).

**Table 1:** Demographic characteristics of AI speaker users and non-users.

Characteristics		User 321(100)	Non-user 3,611(100)
Gender	Male	173(53.90)	1,817(50.30)
	Female	148(46.10)	1,794(49.70)
Age	60–69	7(2.18)	536(14.84)
	50–59	45(14.02)	773(21.41)
	40–49	66(20.56)	768(21.26)
	30–39	90(28.04)	626(17.34)
	20–29	91(28.35)	603(16.70)
	0~19	22(6.85)	305(8.45)
Education	Graduate or above	9(2.80)	95(2.63)
	College	217(67.60)	1,820(50.40)
	High school	86(26.80)	1,371(37.97)
	Middle or elementary school	9(2.80)	321(8.89)
	No education	0(0)	4(0.11)
Marriage	Married	169(52.65)	2,309(63.94)
	Not married	148(46.11)	1,174(32.51)
	etc	4(1.25)	128(3.54)
Job	Administrative management / professional	22(6.85)	258(7.14)
	Office	78(24.30)	597(16.53)
	Sales/service	66(20.56)	679(18.80)
	Technical /labor	36(11.21)	622(17.23)
	Housewife	40(12.46)	610(16.89)
	Student	67(20.87)	630(17.45)
	Inoccupation/etc	12(3.74)	215(5.95)

Note: % in parenthesis.

**Fig 2:**
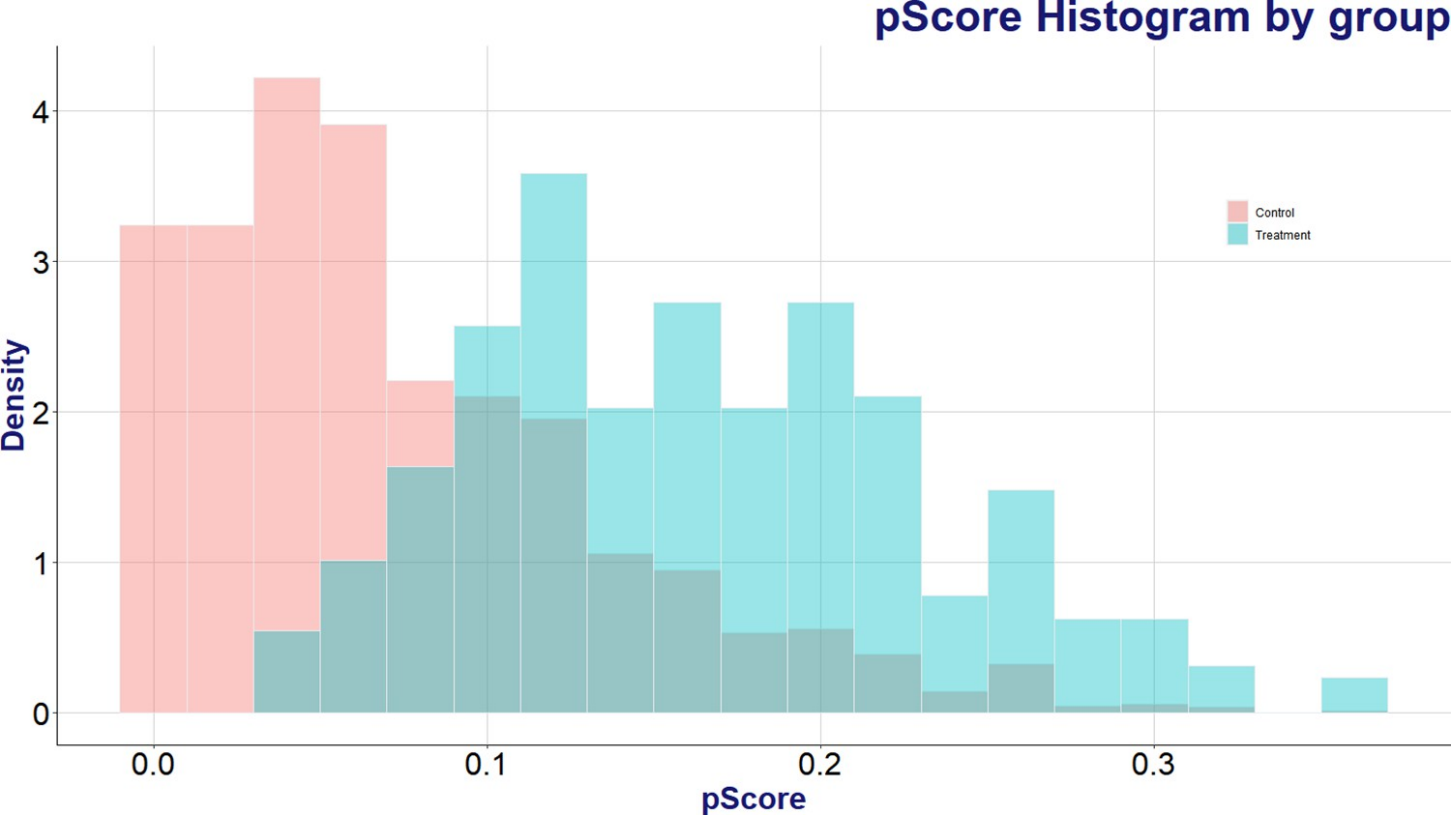
Comparison of propensity score histograms between users and non-users of AI speakers predicted by random forest algorithm.

However, a significant numerical disparity between analysis groups can lead to the problem of statistical bias, where the tendencies of the larger group unduly influence the analysis. Therefore, this study adopted a ‘matching’ statistical method to make the distribution of demographic characteristics between the user group and the non-user group identical and adjust the data. Statistical matching via the Random Forest algorithm was employed to align the distribution of confounding variables between the treatment and control groups. 321 AI speaker users were categorized into a treatment group, while 3,611 non-users formed the control group. A dependent variable was assigned with ’1’ for instances where the product was in use and ’0’ for instances where it was not. Fig 2 illustrates the distribution of propensity scores between the AI speaker user group and the non-user group, as predicted by the random forest algorithm. Given that the criterion for the dependent variable during model training is whether AI speakers are in use or not, the propensity score (depicted in blue) for the user group is observed to be higher than that for the non-user group (depicted in red).

**Fig 3:**
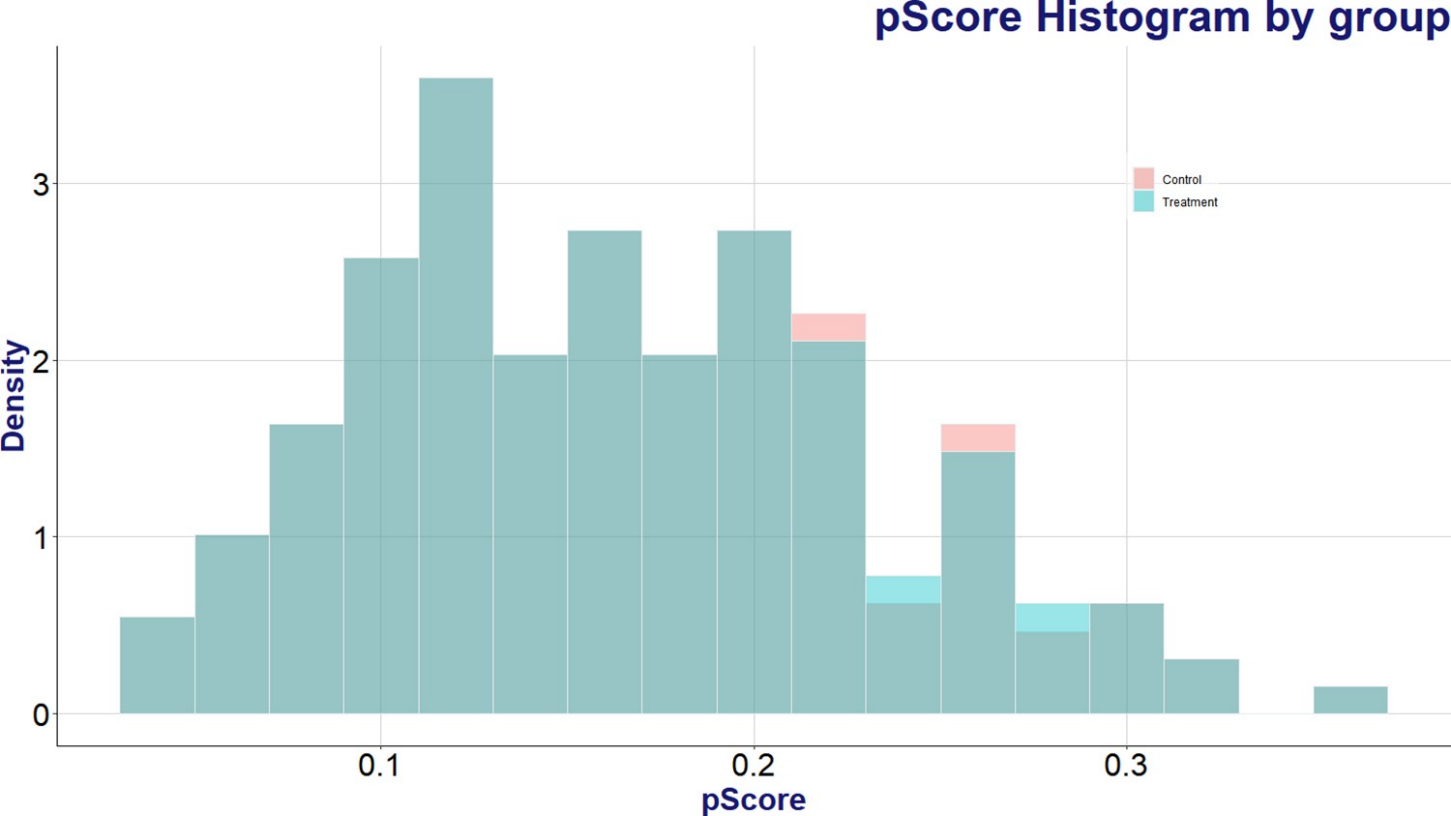
Propensity score histograms of AI speaker users and non-users matched by the nearest neighbor algorithm.

To equate the distribution of propensity scores between the two groups, the nearest-neighbor matching algorithm was employed for data matching. Based on a comparative test, which indicated the highest similarity in propensity scores when the sample sizes for both the user and non-user groups were equal, we configured the sample sizes in a 1:1 ratio for the nearest-neighbor matching algorithm. Fig 2 displays the distribution of propensity scores between the matched AI speaker user and non-user groups. As a result of the matching process, 320 users and 320 non-users with the most closely aligned propensity scores were selected for analysis. A comparison of Figs 2 and 3 reveals that the distribution of non-users, which initially leaned towards lower propensity scores, closely aligns with the user group in Fig 3 post-matching.

### 5.2. Selection of a predictive model for AI speaker acceptance

To develop a predictive model for AI speaker demand, this study performed 10-fold cross-validation using a decision tree model and achieved a cp value of 0.047, representing the lowest cross-validation error rate. The validated decision tree model was then tested with 76 data points, yielding 55 True Positives (TPs) for correctly identifying AI speaker users, 17 True Negatives (TNs) for correctly classifying non-users, 9 False Negatives (FNs) for misclassifying users as non-users, and 47 False Positives (FPs) for incorrectly identifying non-users as users.

The random forest model demonstrated optimal performance when configured with 500 generated trees (ntree = 500) and 7 randomized variables per tree (mtry = 7). Using these hyper-parameters, the confusion matrix revealed 52 TPs and 26 TNs for accurate user and non-user identification, along with 12 FNs and 38 FPs for inaccuracies.

The radial kernel support vector machine model, optimized with sigma = 0.0079 and C = 1, yielded test results of 36 TPs, 12 TNs, 2 FNs, and 26 FPs. The neural network model performed best with 6 hidden nodes (size = 6) and a weight decay metric of 0.3 (decay = 0.3), resulting in a confusion matrix of 42 TPs, 27 TNs, 22 FNs, and 37 FPs.

The XGboost model emerged as the most effective when hyper-parameters were set at nrounds = 200, max_depth = 3, eta = 0.3, gamma = 0, colsample_bytree = 0.8, min_child_weight = 1, and subsample = 1. Its predictive results included 52 TPs, 28 TNs, 12 FNs, and 36 FPs.

Table 2 summarizes the confusion matrix results for all six machine learning algorithms tested for AI speaker demand prediction. Based on metrics such as sensitivity (0.813), F1-score (0.684), and AUC (0.665), the XGboost model is identified as the best predictive model for AI speaker acceptance.

**Table 2:** Performance of models in predicting AI speaker users by machine learning algorithm.

	Sensitivity	F1-score	Precision	Accuracy	AUC
**Decision tree**	0.797	0.634	0.526	0.539	0.505
**Random forest**	0.781	0.676	0.595	0.625	0.624
**Support vector machine**	0.813	0.65	0.542	0.563	0.625
**Artificial neural network**	0.656	0.587	0.532	0.539	0.565
**XGboost**	0.813	0.684	0.59	0.625	0.665

### 5.3. Predictors of AI speaker adoption

In this study, we conducted a Shapley Additive Explanations (SHAP) value analysis to identify significant predictors for the adoption of AI speakers. The SHAP value is a measure of a feature’s contribution across all possible combinations of features and is calculated by weighting and summing the marginal contributions of different feature values. Due to its robustness and interpretability, SHAP values are considered effective for understanding both individual predictions and overall model behavior [74].

**Fig 4:**
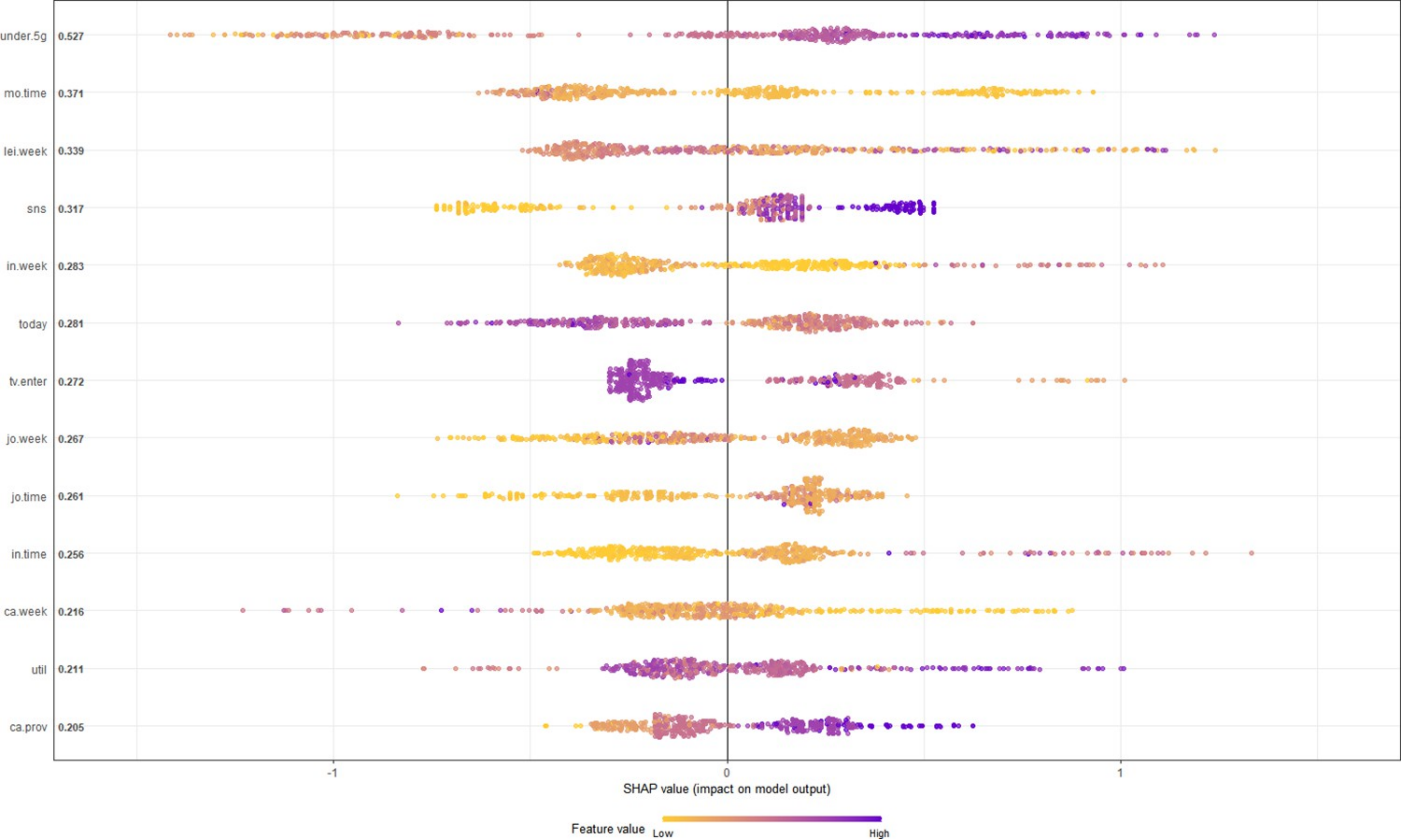
Key predictors of AI speaker acceptance based on SHAP value.

Initially, we assessed 25 variables in ascending order of SHAP values and eliminated the least impactful 12, refining our list to the key 13 variables. Fig 4 visualizes these variables, indicating both their importance and their directional influence on AI speaker adoption. In this figure, each dot represents an individual observation’s SHAP value for a particular variable; the vertical axis displays the variable value, while the horizontal axis represents the corresponding SHAP value. Relative to the centerline at ’0’, positive SHAP values suggest a positive effect on the outcome, whereas negative values indicate the opposite. The color scheme also communicates the variable’s value, with purple representing higher values and yellow representing lower values.

The most significant variables, ranked by their SHAP values, are as follows: ① Understanding of 5G (under.5g), ② Average mobile usage time per day on weekdays (mo.time), ③ Average leisure time per day on weekends (lei.week), ④ Frequency of SNS usage (sns), ⑤ Average internet usage time per day on weekends (in.week), ⑥ Present-oriented life values (today), ⑦ Attitude towards the entertainment value of public broadcasting (tv.enter), ⑧ Average viewing time of comprehensive programming channels on weekends (jo.week), ⑨ Average viewing time of comprehensive programming channels on weekdays (jo.time), ⑩ Average internet usage time on weekdays (in.time), ⑪ Average cable broadcasting viewing time on weekends (ca.week), ⑫ Utility-oriented consumption value (util), and ⑬ Attitude toward sensationalism in cable broadcasting (ca.prov) (refer to Fig 4 for more details).

### 5.4. Relationship between AI speaker acceptance and key predictors

**Fig 5:**
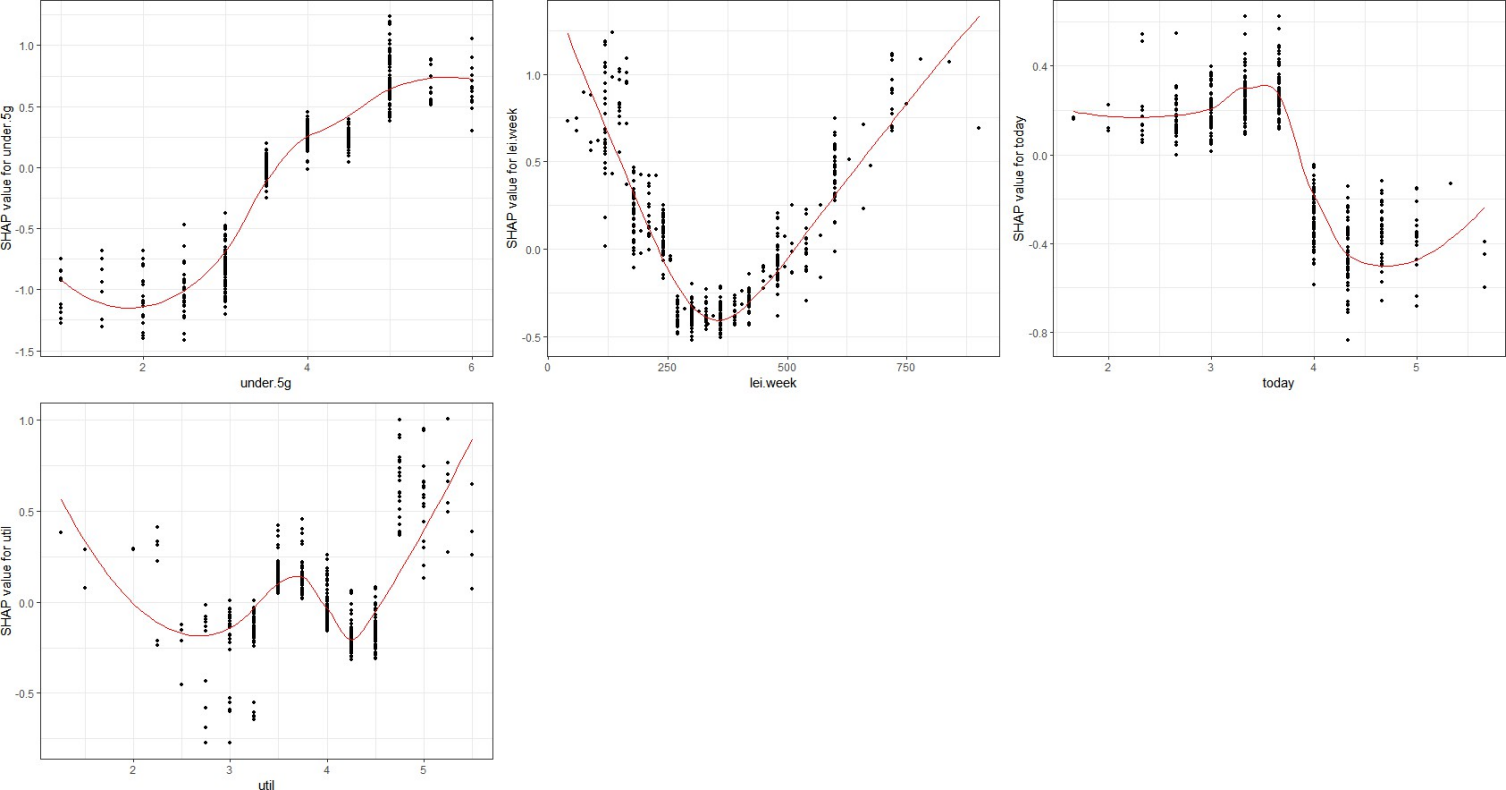
Graphs of SHAP value by key predictors of lifestyle and consumption value.

The most potent predictor for the adoption of AI speakers was the understanding of 5G. Its SHAP value exhibits a stable positive influence on the outcome variable, signifying that AI speaker users generally have a better grasp of 5G technology than non-users. Additionally, average leisure time on weekends has a significant association with AI speaker adoption, with the SHAP value indicating that users are prone to spend over six hours in leisure activities during weekends. Conversely, the value of being present-oriented was negatively correlated with the likelihood of using AI speakers (see Fig 5).

**Fig 6:**
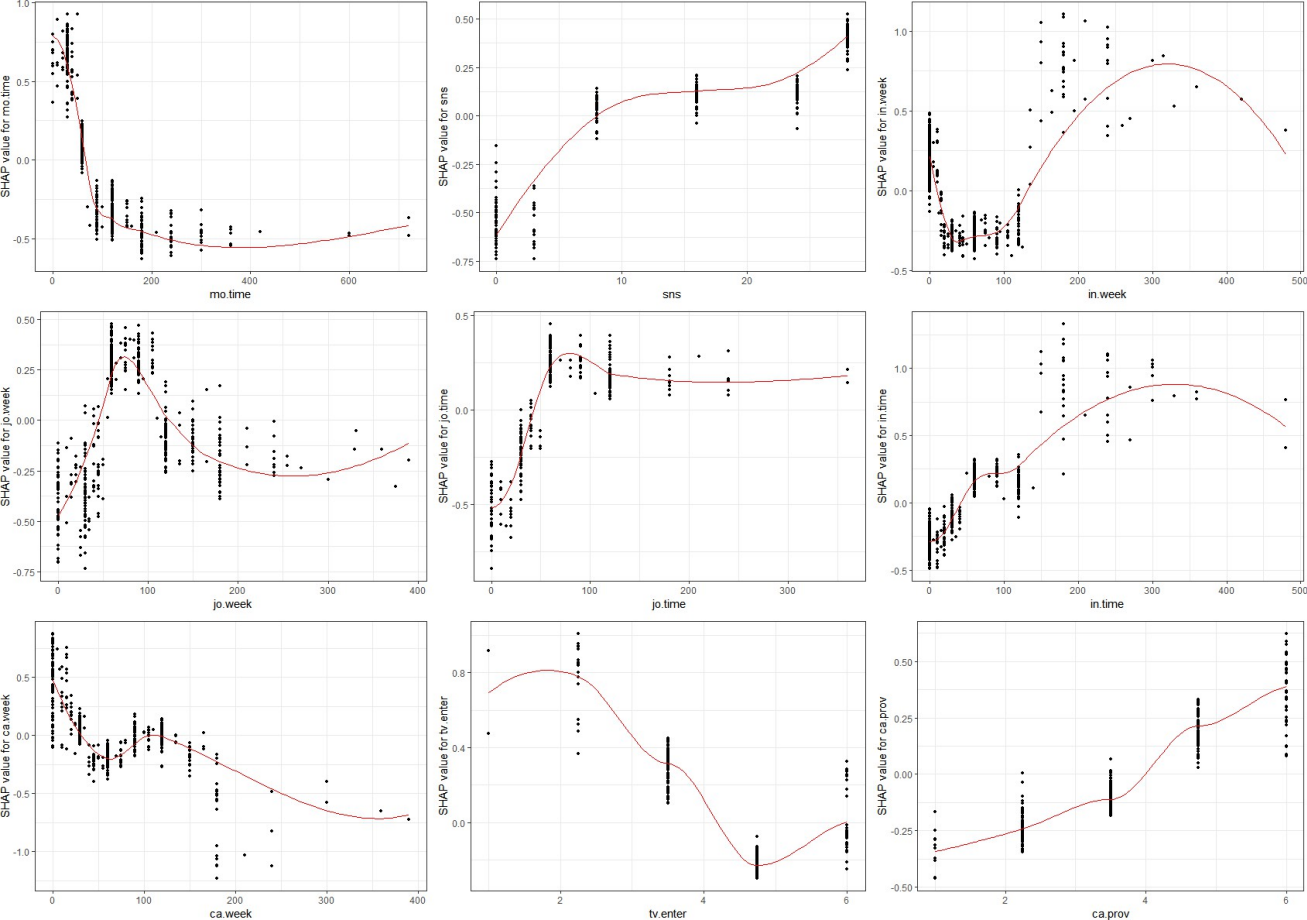
Graphs of SHAP value by key predictors of media.

Media-related factors comprised the largest category, accounting for nine variables. Notably, the SHAP values for weekday and weekend internet usage were positively correlated with the likelihood of adopting AI speakers. Specifically, the weekday internet usage SHAP value increases consistently, suggesting that greater internet usage during weekdays enhances the probability of AI speaker adoption. However, the SHAP value for weekend internet usage initially decreases until approximately 30 minutes before rebounding and stabilizing around the 5-hour mark. Social media usage also exhibited a positive relationship, indicating that frequent social media users are more inclined to use AI speakers. In contrast, mobile usage time during weekdays displayed a declining SHAP value as usage time increased, implying an inverse relationship with AI speaker adoption (see Fig 6).

Additionally, the SHAP value for the average viewing time of comprehensive programming channels showed variable significance based on whether the data was collected during weekdays or weekends. We observed that AI speaker users are more likely to spend extended periods watching these channels on weekdays but limit themselves to 70–80 minutes over the weekend. Moreover, the SHAP value for weekend cable broadcasting viewing time revealed a more intricate trend, increasing within the 50–100 minute range before declining.

Further, the SHAP value associated with utility-oriented consumption showed a complex pattern: initially declining when the consumption value score is low, then rising in the latter half of the scoring range (see Fig 5). This suggests that consumers who place high emphasis on factors like product quality, price, and time-saving are more predisposed to adopt AI speakers.

Lastly, our investigation into the potential interaction effects among the 13 selected key predictors yielded no significant interactions, reinforcing the individual importance of each variable in predicting AI speaker adoption (see S1 Appendix).

## 6. Results for profiling potential consumers of AI speakers

This study employed the XGboost final model to forecast the likelihood of AI speaker adoption among non-users, aiming to profile potential consumers. The model’s predictions ranged from 1.9% to 98.8%. Data were extracted from 234 individuals in the top 10% and 404 in the bottom 10% of predicted usage probabilities. These groups were categorized and compared based on their characteristics.

**Fig 7:**
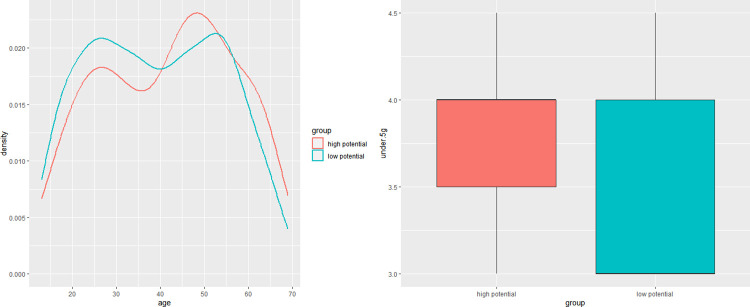
Age distribution and 5G understanding between the potential consumer group and the bottom 10% group for AI speaker.

As shown in Table 3, regarding to demographics, a statistically significant difference in education levels between the groups was also noted; 52.56% of the top group had college education compared to 58.91% in the bottom group (two-tailed, p = .002). In comparison, independent sample Yuen’s t-test results show that the demographic variables of age, income, and expenditure are all statistically insignificant at the 95% confidence interval. However, the results for age and expenditure are very close to the p-value of 0.05, which requires more careful interpretation. The average age of potential consumers in the top 10% of AI speakers is 41.68 years old, slightly higher than the bottom 10% (M = 39.19). Actually, we can see that the top 10% of AI speakers has a higher proportion of 45-50-year olds and 60–65 year olds than the bottom 10%. This suggests that people in these age groups are more likely to be the primary potential consumers. The graph on the left in Fig 7 provides a visual representation of this result. Furthermore, the spending level of the top 10% group is somewhat higher than the bottom 10% group (KRW 1,016,000), on average, compared to KRW 761,000. According to the Chi-square test, the difference in occupation between the two groups was not statistically significant (see Table 4).

**Table 3:** Fisher’s exact test results for gender, education, and occupation variables.

**Division**	**Gender**						Grand total	df	*p*
	Male			Male					
Potential consumer	123			111			638	1	1
Bottom 10%	213			191					
Total	336			302					
**Division**	**Education**						Grand total	df	*p*
	Elementary school or below	Middle school	High school		College	Graduate or above			
Potential consumer	0	21	90		108	15	638	4	.002
Bottom 10%	0	26	140		231	7			
Total	0	50	246		353	22			

**Table 4:** Chi-square test results for the occupation variable.

**Division**	**Job**							df	x^2^	Cramer’s V
	Administrative /professional	Office	Sales/ service	Technical /labor	House wife	Student	Inoccupation /etc			
Potential consumer	16	35	37	43	36	50	17	6	8.02	0.24
Bottom 10%	27	76	73	66	62	87	13			
Total	43	101	110	109	98	137	30			

Analysis of the 13 key predictive variables mostly aligned with the SHAP value findings, barring three variables. The potential consumer group was notably more active on social media and spent more time watching comprehensive programming channels. Specifically, they used social media approximately 14.88 times per month, almost five times more frequently than the bottom group (M = 9.31, SD = 14.23); Tw(636) = 4.93, p < .001, δ = 0.4. Moreover, this group spent more time watching comprehensive programming channels on both weekdays and weekends compared to the bottom group, reinforcing the SHAP value analysis for weekday viewing but complicating the interpretation for weekend viewing. The potential consumer group also exhibited tendencies to use the internet more during weekdays and watch more cable TV during weekends than the bottom 10%. They spent an average of 43 minutes on the internet on weekdays and watched about 70 minutes of cable TV on weekends, both higher than the bottom group. Interestingly, their mobile usage on weekdays was lower compared to the bottom group. Attitudinal differences were observed between the groups concerning public broadcasting and cable TV. The potential consumer group rated public broadcasting as less entertaining but found cable TV to be more sensational.

Understanding of 5G emerged as a significant factor among potential consumers, corroborating its role as the strongest predictor in the SHAP value analysis. They scored an average of 4 in understanding 5G, significantly higher than the bottom 10% and the general respondents. This variable had the highest effect size among all key variables. The right-hand side of Fig 7 visualizes the difference in understanding of 5G between the potential consumer group and the bottom 10%. The potential consumer group spent an average of about 6 hours per day on leisure on weekends (SD = 268.27), which was 50 minutes longer than the bottom 10% group’s average of about 5 hours and 10 minutes (SD = 131.37); Tw(170.54) = 2.44, p = .02, ξ = 0.18.

Finally, the potential consumer group of AI speaker was found to have higher levels of utility-oriented consumption values than the bottom group (see Table 5). However, of the total 13 key predictors, present-centered life values and weekend internet time did not reveal statistical significance between the two groups within the 95% confidence interval.

**Table 5:** Independent sample Yuen’s t-test results for AI speaker potential consumers and bottom 10% group.

Division		Potential consumer group (n = 234)		Bottom 10% group (n = 404)		95% CI for Mean Difference	tw	df	*p*	δ (ξ)
M	SD	M	SD							
Demographic	Age	41.89	18.27	39.09	18.16	-0.24, 6.04	1.81	297.01	.07	-
	Income	154.26	220.33	175.46	228.96	-64.92, 14.84	1.23	300.51	.22	-
	Expenditure	101.6	76.1	92.9	79.11	-0.55, 29.56	2.37	443.56	.06	-
Lifestyle	Understanding of 5G	4	0.96	3.17	0.92	0.69, 0.99	10.84	295.41	0	0.95
	Average leisure time on weekends	356.33	268.27	312.9	131.37	8.57, 81.77	2.44	170.54	.02	(0.18)
	Present-oriented life values	3.57	0.97	3.6	0.68	-0.17, 0.09	0.56	245.35	.58	-
Media	average mobile usage time on weekdays	62.93	53	86.23	51.1	-36.05, -18.68	6.2	241.93	0	0.58
	Frequency of SNS usage	14.88	19.08	9.31	14.23	4.05, 9.85	4.72	269.09	0	0.42
	Average Internet usage time on weekdays	43.03	62.79	27.18	38.61	0.71, 21.65	2.11	194.83	.03	0.19
	Average internet usage time on weekdays	31.76	43.02	27.86	37.58	-10.86, 6.94	0.43	223.31	.66	-
	Average viewing time of comprehensive programming channels on weekdays	62.87	45.02	43.64	38.25	11.93, 22.76	6.3	297.48	0	0.58
	Average comprehensive programming channels viewing time per day on weekends	81.35	44.13	57.01	57.57	18.12, 33.97	6.47	352.6	0	0.53
	Average cable TV viewing time on weekends	70.98	74.05	54.02	41.98	12.77, 31.65	4.64	198	< .001	(0.28)
	Attitude toward entertainment on public broadcasting	4.02	1.37	4.7	0	-0.83, -0.56	10.32	141	0	(0.54)
Consumption value and attitude	Attitude toward sensationalism of cable broadcasting	4.04	2.11	3.57	1.39	0.26, 0.8	3.88	252.57	< .001	(0.24)
	Utility orientation	3.97	0.84	3.81	0.61	0.02, 0.26	2.34	242.64	.02	(0.15)

## 7. Conclusion

Using data from the 2019 Media & Consumer Research (MCR) survey conducted by the Korea Broadcasting and Advertising Corporation, this study builds AI speaker adoption prediction model using five machine learning techniques: decision trees, random forests, support vector machines, artificial neural networks, and XGboost.

In the AI speaker acceptance prediction analysis, the XGboost model was selected as the final model with the best performance among the five models. The main predictors included understanding of 5G, average mobile usage per day on weekdays, average leisure time per day on weekends, frequency of social media usage, average internet usage per day on weekends, present-oriented life values, attitudes toward the entertainment value of public broadcasting, average total channel viewing per day on weekends, average total channel viewing per day on weekdays, average internet usage per day on weekdays, average cable broadcasting viewing per day on weekends, utility-oriented consumption values, and attitudes toward sensationalism in cable broadcasting. Also, the results identified key demographic characteristics and consumer behaviors that influence future AI speaker adoption. Individuals between the ages of 45–50 and 60–65 were identified as potential consumers of AI speakers, as they are frequent users of social networking services and prefer comprehensive programming channels. Potential AI speaker consumers have high internet usage during the week and cable viewing on the weekends. They also have a high level of understanding of 5G technology and spend more time on leisure on the weekends. These results show that AI speaker ad marketing practitioners need to take a strategic approach to media planning, prioritizing social media and aggregation channels, and designing ad marketing concepts and messages that target relatively older audience. Additionally, they can also consider a “day-of-the-week” media strategy, where they differentiate the quantity of their ads on the internet on weekdays and on cable on weekends respectively.

The study contributes both academically and practically to the literature, identifying new variables associated with the acceptance of new technology products and generating findings beneficial for the industry. To navigate this rapidly changing environment, industry stakeholders require effective advertising and marketing strategies. In this regard, this study utilizes consumer data-driven analyses to discover new variables not covered by existing models, demonstrating that factors such as consumer values, attitudes, and lifestyles significantly affect IoT product acceptance. Moreover, this study provides invaluable information about target consumer behaviors, such as media usage, attitudes toward media, consumption values, and leisure activities. Such data is often challenging to acquire through individual company resources and day-to-day business operations. Consequently, the study is expected to make a practical contribution to the development of industry advertising and marketing strategies.

Furthermore, the academic value of this study lies in its empirical validation of machine learning techniques as viable research methodologies in the social sciences, particularly within the realms of digital advertising and marketing. [75] highlight the challenges traditional methods face in analyzing interactive communication in digital media and advocated for the adoption of new analytic techniques. They critiqued the excessive focus on quantitative methodologies in Korea’s digital media advertising research and called for the inclusion of qualitative research enriched by inductive reasoning. However, the universal validity of qualitative research has long been a subject of debate, due in part to the limited sample sizes and open-ended research methodologies. Machine learning techniques, capable of refining and analyzing large data sets, stand out as a methodology that can address these limitations. Specifically, machine learning does not rely on the data assumptions often needed in traditional statistical analyses, thus offering greater flexibility for empirical researchers.

Despite these contributions, the study has its limitations. First of all, it focuses more on the consumers of new technology rather than the technology itself. Although the study aimed to include a comprehensive set of variables, questions remain about the sufficiency of the independent variables analyzed. Only eight lifestyle variables were selected as independent variables through a two-step preliminary research process, leaving open the possibility that additional factors could enrich the analysis. This study leveraged the MCR public survey data to access high-quality, rich consumer information, but the sample size of 321 AI speaker users out of 3,922 respondents is relatively small. This limitation could affect the reliability of the machine learning model, depending on the data partitioning into training and test sets. Moreover, this study has produced useful results as an initial step for look-alike targeting strategies in advertising marketing, but due to the limitation of secondary analysis using existing data, it has not reached the stage of suggesting detailed advertising marketing strategies. Therefore, we hope that future studies will be conducted to secure and analyze sufficiently large and rich consumer data to increase the reliability of the research results and suggest more realistic and in-depth advertising marketing strategies.

## Supporting information

S1 Appendix Tables for the items measuring variables and beeswarm plots of SHAP values for main predictors. (DOCX)
