# Supplementary material for: Profiling the AI speaker user: Machine learning insights into consumer adoption patterns
Source: PLoS One. 2024 Dec 18;19(12):e0315540. doi: 10.1371/journal.pone.0315540 (PMC11654937; doi:10.1371/journal.pone.0315540)

**Appendix**

Table A1. Items for measuring consumption values

| **Variable** | | **Detail** |
| --- | --- | --- |
| Consumption community value | Social justice orientation | When purchasing a product, I tend to consider the corporate image. |
|  |  | I believe that a company that contributes more to society is a better company. |
|  |  | The business ethics of companies that produce or sell products often influence which products I buy. |
|  | Eco-friendly orientation | We can accept inconveniences for the sake of the environment. |
|  |  | We can accept inconveniences for the sake of the environment. |
|  |  | I feel responsible for environmental issues. |
|  |  | I can change my consumption habits for the environment. |
|  |  | Even if it is expensive, I am willing to pay more for the environment. |
| product attribute value | Utility orientation | I buy products with good performance and quality rather than design. |
|  |  | If I can save time, I can pay a higher price |
|  |  | I tend to carefully consider credit card and coupon discount benefits. |
|  |  | I buy products that are cheap even if the design or quality is not great. |
|  | Safety orientation | I like to look at products in stores first and then purchase them online. |
|  |  | I tend to continue purchasing brands I have experience with. |
| individual-oriented value | Aesthetic orientation | I buy products with excellent design even if the price is high. |
|  |  | I buy products with excellent design even if the quality is not excellent. |
|  | Innovation-orientation | I think I like creative companies or brands that are differentiated from others. |
|  | Pleasure orientation | Shopping is the joy of my life. |
|  |  | I love seeing new product demonstrations and displays in stores. |
| consumer subjective value | Autonomy | People often buy products recommended by salespeople. |
|  |  | People often buy products recommended by people around them. |
|  | Self-expression orientation | I am willing to pay more to purchase a product before others. |
|  |  | I believe that the products I buy express who I am. |
|  |  | I love finding out the latest products or brands that other people don't know about yet. |
| Other-oriented value | Show-off orientation | I tend to buy famous brand products even if they are expensive. |
|  |  | Which brand you buy is related to your status. |

Table A2. Items for impulsivity and behavioral tendencies

| **Variable** | **Detail** |
| --- | --- |
| impulsivity and behavioral tendencies | When purchasing a product, I compare thoroughly before purchasing. |
|  | I often read packaging, brochures, and information on the Internet to learn more about the brands I purchase. |
|  | I often look in magazines or on the Internet for products or services that interest me. |
|  | When I watch a home shopping channel, I feel the urge to buy something |
|  | I often impulsively buy unplanned items |

Table A3. Items for measuring lifestyle

| **Variable** | | **Detail** |
| --- | --- | --- |
| leisure | Leisure time | Average leisure time on weekdays (minutes) |
|  |  | Average leisure time on weekends (minutes) |
|  | Alone leisure orientation | It is better to spend my leisure time alone. |
|  |  | I feel more comfortable eating out or watching a movie alone than with other people. |
|  |  | I prefer watching TV alone than with my family |
| values ​​about life | Traditional Confucianism | During my free time, I mainly engage in activities for self-development. |
|  |  | Marriage is something people should do. |
|  | Individualism | I don't think getting married necessarily means having children. |
|  |  | Family is important, but you have to think of yourself first. |
|  | Hedonism | I want to save up a few months' worth of my salary and go on a trip I will remember for the rest of my life. |
|  |  | Being happy in the present moment is more important than preparing for the future. |
|  |  | It's not a waste of money to invest in my favorite hobby. |
| Attitude toward 5G | Understanding of 5G | We are well aware of the changes that the popularization of 5G will bring. |
|  |  | With the commercialization of 5G, changes can be felt in many fields. |
|  | Favorability of 5G | As the 5G network expands, the quality of life will improve. |
|  |  | I am interested in products combined with 5G. |
|  |  | I am willing to purchase products combined with 5G even if they are more expensive. |
| Life satisfaction | | I am satisfied with my life. |
|  |  | My life is close to my ideal life. |
|  |  | Even if I were born again, I want to live my current life again. |
|  |  | So far, I have everything I thought was important in my life. |
|  |  | I am satisfied with many conditions in my life. |

Table A4. Items for measuring media use and attitudes

| **Variable** | | **Detail** |
| --- | --- | --- |
| Media use | Public broadcasting | Average viewing time per day on weekdays (minutes) |
|  |  | Average viewing time per day on weekends (minutes) |
|  | Comprehensive Programming Channel | Average viewing time per day on weekdays (minutes) |
|  |  | Average viewing time per day on weekends (minutes) |
|  | Cable TV | Average viewing time per day on weekdays (minutes) |
|  |  | Average viewing time per day on weekends (minutes) |
|  | Radio | Average listening time per weekday (minutes) |
|  |  | Average listening time per day on weekends (minutes) |
|  | DMB | Average viewing time per day on weekdays (minutes) |
|  |  | Average viewing time per day on weekends (minutes) |
|  | Internet | Average usage time per day on weekdays (minutes) |
|  |  | Average usage time per day on weekends (minutes) |
|  | Mobile | Average usage time per day on weekdays (minutes) |
|  |  | Average usage time per day on weekends (minutes) |
|  | News paper | Average reading time per day on weekdays (minutes) |
|  |  | Average reading time per day on weekends (minutes) |
| SNS | | Monthly average frequency of SNS use |
| Attitude toward media | Entertainment | Public broadcasting |
|  |  | Comprehensive Programming Channel |
|  |  | Cable TV |
|  |  | Radio |
|  |  | DMB |
|  |  | PC |
|  |  | Mobile |
|  |  | News paper |
|  | Reliability | Public broadcasting |
|  |  | Comprehensive Programming Channel |
|  |  | Cable TV |
|  |  | Radio |
|  |  | DMB |
|  |  | PC |
|  |  | Mobile |
|  |  | News paper |
|  | Fairness | Public broadcasting |
|  |  | Comprehensive Programming Channel |
|  |  | Cable TV |
|  |  | Radio |
|  |  | DMB |
|  |  | PC |
|  |  | Mobile |
|  |  | News paper |
|  | Information | Public broadcasting |
|  |  | Comprehensive Programming Channel |
|  |  | Cable TV |
|  |  | Radio |
|  |  | DMB |
|  |  | PC |
|  |  | Mobile |
|  |  | News paper |
|  | Sensationalism | Public broadcasting |
|  |  | Comprehensive Programming Channel |
|  |  | Cable TV |
|  |  | Radio |
|  |  | DMB |
|  |  | PC |
|  |  | Mobile |
|  |  | News paper |

Figure A1. Beeswarm plot of SHAP values for the top 7 predictors


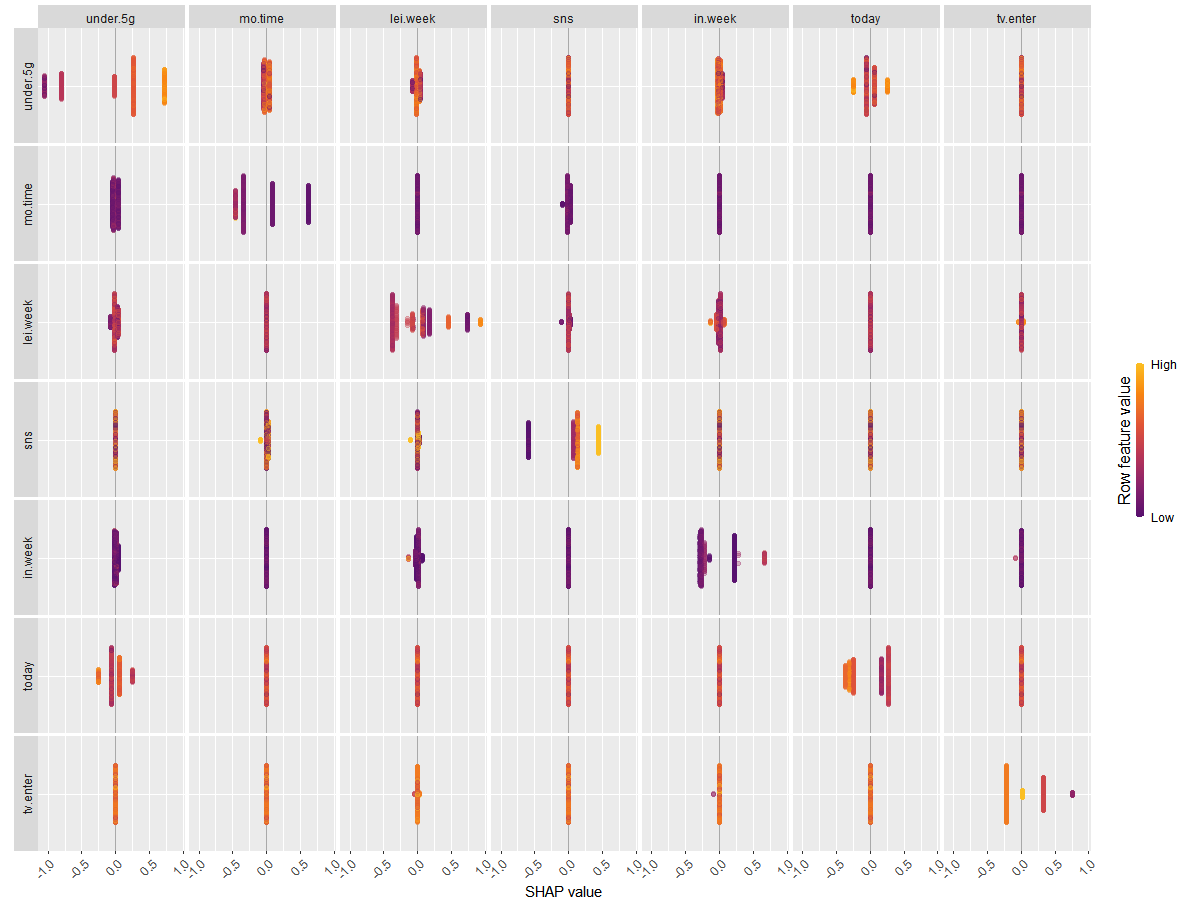


Figure A2. Beeswarm plots of pairs of highly interacting variables


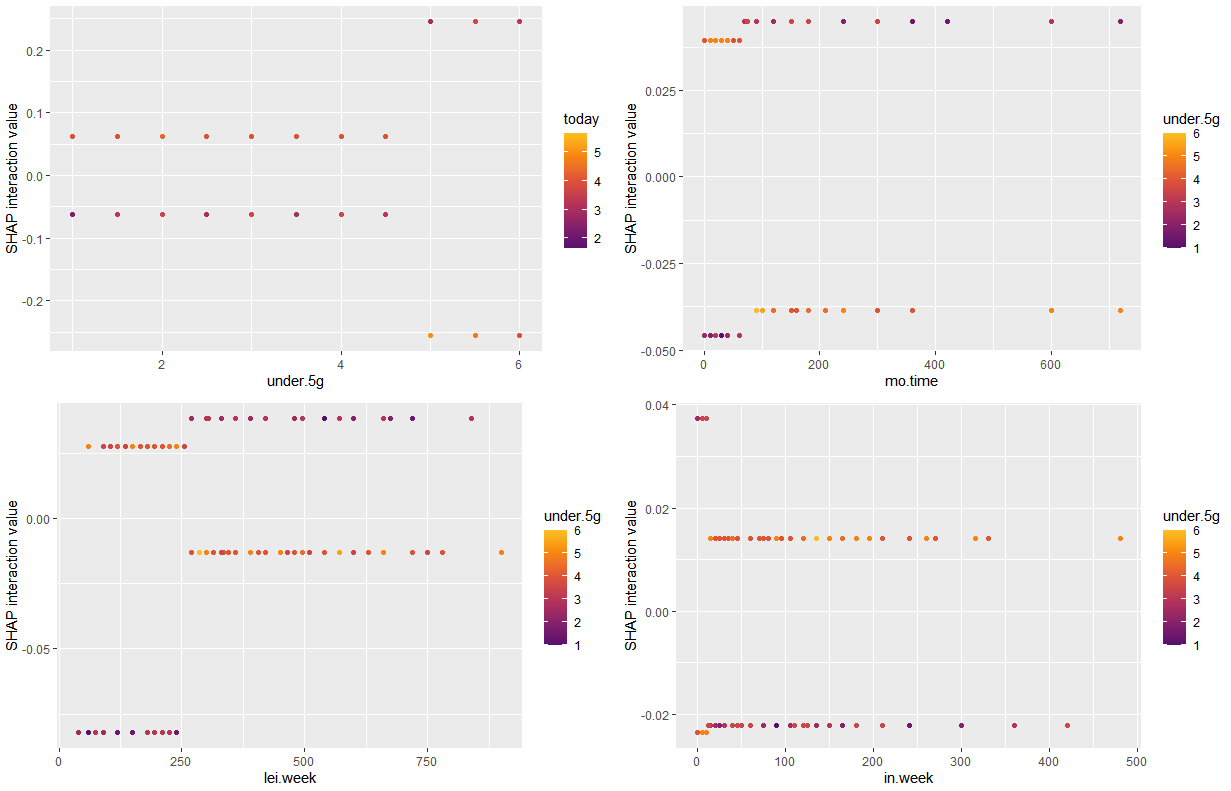

Supplement: S1 Appendix — (DOCX) [file pone.0315540.s001.docx]
